# Disrupting the Interplay between Programmed Cell Death Protein 1 and Programmed Death Ligand 1 with Spherical Nucleic Acids in Treating Cancer

**DOI:** 10.1021/acscentsci.2c00717

**Published:** 2022-08-31

**Authors:** Liyushang Chou, Cassandra E. Callmann, Donye Dominguez, Bin Zhang, Chad A. Mirkin

**Affiliations:** ^†^Interdisciplinary Biological Sciences Graduate Program, ^‡^International Institute for Nanotechnology, and ^§^Department of Chemistry, Northwestern University, Evanston, Illinois 60208, United States; ∥ Feinberg School of Medicine, Northwestern University, Chicago, Illinois 60611, United States

## Abstract

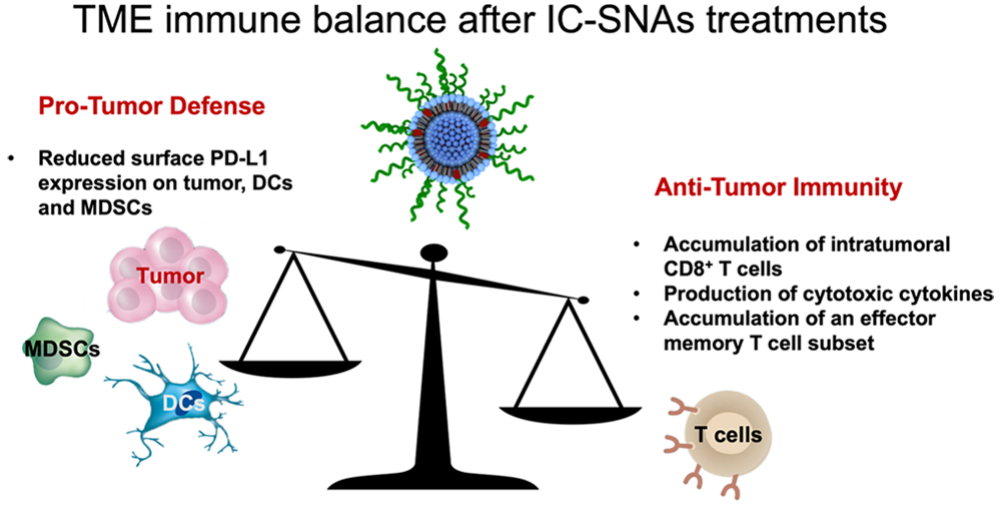

Disrupting the interplay between programmed cell death protein 1 (PD-1) and programmed death ligand 1 (PD-L1) is a powerful immunotherapeutic approach to cancer treatment. Herein, spherical nucleic acid (SNA) liposomal nanoparticle conjugates that incorporate a newly designed antisense DNA sequence specifically against PD-L1 (immune checkpoint inhibitor SNAs, or IC-SNAs) are explored as a strategy for blocking PD-1/PD-L1 signaling within the tumor microenvironment (TME). Concentration-dependent PD-L1 silencing with IC-SNAs is observed in MC38 colon cancer cells, where IC-SNAs decrease both surface PD-L1 (sPD-L1) and total PD-L1 expression. Furthermore, peritumoral administration of IC-SNAs in a syngeneic mouse model of MC38 colon cancer leads to reduced sPD-L1 expression in multiple cell populations within the TME, including tumor cells, dendritic cells, and myeloid derived suppressor cells. The treatment effectively increases CD8^+^ T cells accumulation and functionality in the TME, which ultimately inhibits tumor growth and extends animal survival. Taken together, these data show that IC-SNA nanoconstructs are capable of disrupting the PD-1/PD-L1 interplay via gene regulation, thereby providing a promising avenue for cancer immunotherapy.

## Abstract 2

Spherical nucleic acids that incorporate a newly designed antisense DNA sequence against PD-L1 are presented as a genetic approach to disrupting the PD-1/PD-L1 axis in cancer immunotherapy.

## Introduction

Cancer remains a leading cause of death worldwide, necessitating the continued study of disease etiology and the development of new treatment options.^1,2^ Owing to an increased understanding of the hallmarks of many cancers (e.g., uncontrolled cell proliferation triggered by genetic mutations, rewired cell signaling, dysregulated metabolism, and increased immune evasion mechanisms),^3,4^ cancer treatment has shifted away from surgery and radiotherapy toward targeted chemo- and immunotherapies. A particularly promising immunotherapeutic strategy involves disrupting the interplay between programmed cell death protein 1 (PD-1) and programmed death ligand 1 (PD-L1) within the tumor microenvironment (TME).^5−8^ PD-1 is a type I transmembrane glycoprotein found on the surface of CD8^+^ T cells,^9^ while PD-L1 is overexpressed on the surface of cancer cells, antigen-presenting cells (APCs), macrophages, and myeloid-derived suppressive cells (MDSCs) in the TME.^10^ The binding of PD-L1 to PD-1 initiates a negative signal cascade that inhibits T cell activation and decreases T cell cytotoxicity. Furthermore, the upregulation of PD-L1 on multiple cell types causes the inadequate priming of CD8^+^ T cells and leads to decreased infiltration of activated T cells into the TME. Cumulatively, this reduces the cytotoxic effect of T cells on tumors.^11^

Because the PD-1/PD-L1 pathway is a dominant regulator of cancer immune response, blockade therapies that target this pathway have been developed and implemented in the clinic.^7,8,10^ For example, monoclonal antibodies that bind with high affinity to PD-1 on the surface of T cells act as competitive inhibitors of PD-L1. Therapeutic antibodies (commonly referred to as checkpoint inhibitors) against PD-1 have been shown to cause durable and persistent tumor regression when administered to patients with treatment-refractory solid tumors.^12−16^ However, 40–60% of patients do not respond to single-antibody treatment alone.^17−22^ Therefore, antibody-based checkpoint inhibitors that bind PD-L1 are often used in conjunction with those that bind PD-1 to block receptor signal transduction.^23−28^ Despite the success of combination checkpoint inhibitor antibody therapies, many patients acquire resistance to such treatments,^29^ experience significant adverse off-target effects, and/or develop complement-dependent cytotoxicity^30^ from long-term systemic use.^31−34^ Moreover, antibodies do not have access to the intracellular compartments.^35^ Indeed, PD-L1 exists in multiple forms and in different cellular compartments (e.g., transmembrane-anchored, cytosolic, nucleic, and soluble, circulating variants; they also exist as mRNA transcripts in various cells as a self-defense mechanism for potential autophagy) and is involved in multiple immunosuppression pathways, both site-specifically and systemically.^36−39^ Thus, additional effective blockade options are needed. In this regard, gene regulation strategies that decrease the overall expression of PD-L1 are considered a promising means for blocking immune checkpoints.^21,35−43^

Spherical nucleic acids (SNAs) are nanostructures consisting of spherical nanoparticle cores with densely packed and highly oriented oligonucleotides on their surfaces; they have been proven to be extremely useful in biomedicine.^44−50^ Due to their unique architecture, SNAs are more efficiently and rapidly taken up by cells (over 60 types to date, via scavenger receptor-mediated endocytosis^51,52^) as compared to linear oligonucleotides of the same sequence. SNAs also are more resistant to nuclease degradation and have extended circulation half-lives *in vivo* relative to linear nucleic acids,^53,54^ due to the dense arrangement of oligonucleotides on the SNA surface. In addition, the cooperative binding of nucleic acids on SNAs to complementary mRNA targets increases the binding affinity by 1–2 orders of magnitude relative to their linear nucleic acid counterparts under identical conditions.^44,45,55^ Although SNAs are initially taken up via endosomal pathways, a meaningful fraction escape^46−48,56^ and have been shown to act as potent gene regulation^57,58^ agents in the cytosol *in vivo*(50) and in clinical trials.^59^ Importantly, the SNAs in such studies exhibited minimal cytotoxicity and immunogenicity, making them attractive for use as medicines.

Herein, a new anti-PD-L1 antisense DNA sequence was designed, and the SNA architecture was leveraged to deliver this sequence into MC38 colon cancer cells and knockdown PD-L1 expression. These SNAs, designated immune checkpoint inhibitor SNAs (IC-SNAs), were investigated for their ability to regulate the expression of PD-L1 level *in vitro* and *in vivo* as well as for their ability to act as potent anticancer therapies.

## Results and Discussion

### Design of IC-SNAs

**Scheme 1:**
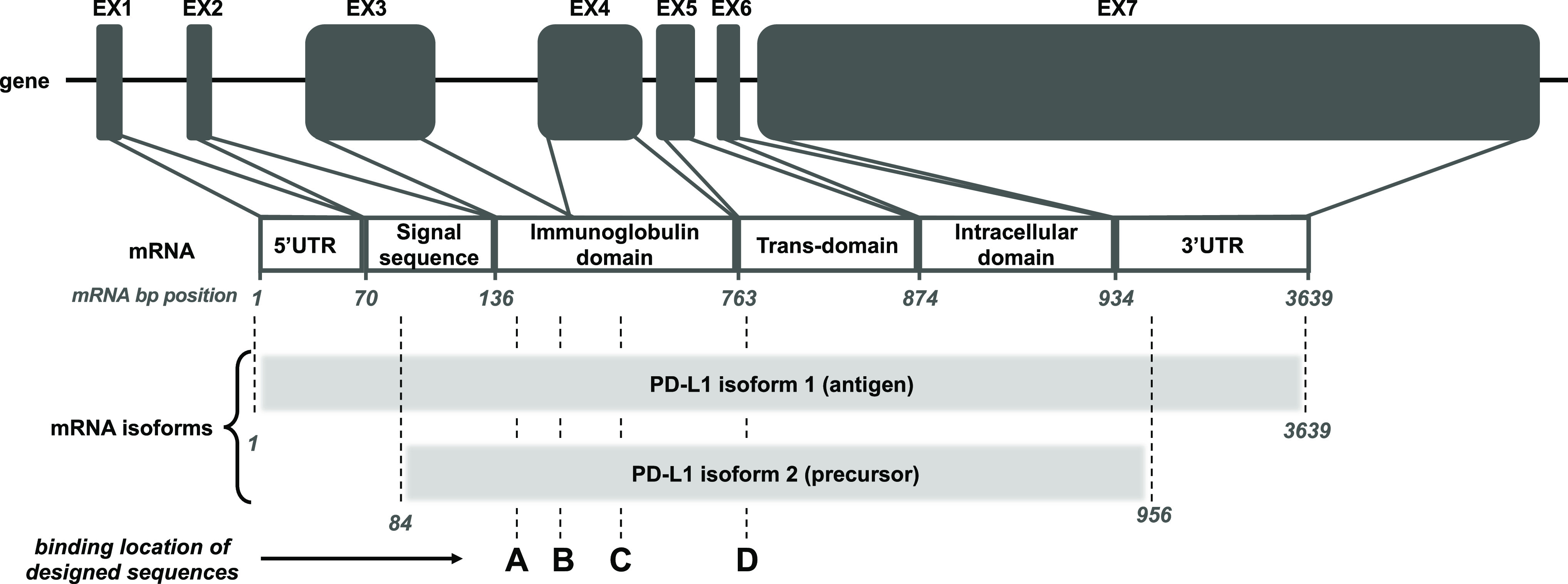
Targeting Sequence Design Rationale The mouse PD-L1 gene (CD274) is located on chromosome 19, which is composed of 7 exons with introns in between. The gene transcript mRNA for CD274 encodes a full script at a length of 3639 base pairs (bp) with six domains in total: 5′ UTR, signal sequence, immunoglobulin (immunoglobulin V-like and C-like), transmembrane, intracellular, and 3′ UTR. The mRNA full scripts adopt mainly two variant isoforms, which can be subsequently translated into PD-L1 antigen and precursor. Both isoforms have full immunoglobulin, transmembrane, and intracellular domains. Therefore, the interactions of these PD-L1 isoforms with their receptors inhibit T cell activation and cytokine production. To target the entire PD-L1 population, test sequences (15 bps in length) were designed to be complementary to mouse PD-L1 (mPD-L1) mRNA in locations such as the mRNA base pair positions 148 (A), 178 (B), 344 (C), and 766 (D).

In order to target PD-L1 using antisense DNA, oligonucleotide sequences capable of knocking down the expression of mouse PD-L1 (mPD-L1) were designed (Scheme 1) using the mPD-L1 mRNA data available from the NCBI GeneBank.^60,61^ The full script of the mRNA sequence transcribed from the mPD-L1 (CD274) gene is comprised of six domains: 5′ UTR (untranslated region), signal sequence, immunoglobulin, transmembrane, intracellular, and 3′ UTR. Given alternative splicing mechanisms for mRNA editing before translation, PD-L1 mRNAs mainly adopt two isoforms in mice. Despite differences in the signal sequence and the 3′ and 5′ UTR regions, both variants contain immunoglobulin, transmembrane, and intracellular domains in their transcripts. Therefore, to target as many PD-L1 protein isoforms as possible at an mRNA level, the oligonucleotide sequences employed herein were designed to target the overlapping regions of the mRNA.

To reduce nonspecific targeting, a BLAST analysis (NCBI Basic Local Alignment Search Tool) was run and screened for sequence homogeneity. Subsequently, to identify a specific binding region, the sequences were designed with the goal of either: (1) recruiting RNase H for targeted degradation or (2) sterically hindering ribosome movement down the translating strand. In the case of the RNase recruitment model, the designed sequence must be able to bind the RNase H cleavage pocket, which is ∼18 base pairs (bp) in length^62−65^ (Scheme S1A). In the latter case, the sequence must be designed to halt ribosome translation via steric hindrance by hybridizing tightly to an accessible region on the target mRNA. Hence, sequence binding should not occur in regions where protein machineries, such as poly-A-binding protein, translation initiation factors, and ribosomes, bind during translation (Scheme S1B). Moreover, the sequence must hybridize tightly to the mRNA to halt translation; thus, the accessibility of the single-strand base pair position and the melting temperature of the hybrid are other factors that were taken into consideration. With these constraints in mind, the sequences were designed by initially simulating a folding process for mPD-L1 mRNA with the Mfold Web Server^66,67^ to generate an accessibility mapping profile using the RNA folding tool. With input of mPD-L1 mRNA, *Mus musculus* CD274 antigen (Cd274), and mRNA NM_021893.3 from NCBI GenBank, the regions with the highest accessibility for hybridization were identified (Scheme 1, Figure S1).

### PD-L1 Knockdown by Designed Sequences with Transfection Agents

**Figure 1:**
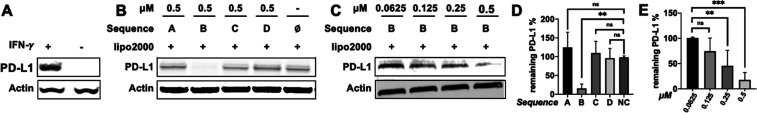
PD-L1 knockdown by antisense DNA sequences. (A) PD-L1 expression level in the MC38 cell line following IFN-γ stimulation. (B) Evaluation of the ability of the designed sequences (A–D) to inhibit PD-L1 expression (sequences A–D, ϕ = untreated) using standard transfection protocols. Cell lysates were harvested after 48 h incubation, and PD-L1 protein level was measured using a Western blot analysis (*n* = 4, representative example shown in panel). (C) Concentration-dependent knockdown of PD-L1 expression in MC38 cells by sequence B (*n* = 4, representative example shown in panel). Actin was used as a standard control to ensure that an equal amount of protein lysates were loaded to each lane. (D) Statistical analysis of reduction in PD-L1 by sequences shown in Figure 1B (*n* = 4). (E) Statistical analysis of concentration-dependent knockdown as shown in Figure 1C (*n* = 4). Statistical analysis was performed using a one-way ANOVA, where “**” *p* < 0.01; “***” *p* < 0.001; and “****” *p* < 0.0001.

Based on these design rules, four antisense oligonucleotide sequence candidates were identified and synthesized (sequence A: 5′-AGT CCT TTG GAG CCG-3′, sequence B: 5′-TGA CGT TGC TGC CAT-3′, sequence C: 5′-AGC TGG TCC TTT GGC-3′, and sequence D: 5′-GAT GTG TTG CAG GCA-3′, sequence positions are labeled in Scheme 1), and subsequently their knockdown efficacy in cells was investigated. In MC38 colon cancer cells cultured in RPMI-1640 media, PD-L1 expression was induced via incubation with interferon-gamma (IFN-γ) (20 ng/mL) overnight (Figure 1A). Compared to the other candidate sequences, sequence B achieved the greatest knockdown of total PD-L1 expression when Lipofectamine 2000 was used as the transfection agent (Figure 1B); knockdown was achieved in a concentration-dependent manner (Figure 1C). Therefore, sequence B was used in all experiments involving SNA formulations.

### PD-L1 Reduction with IC-SNA Treatments *In Vitro*

**Figure 2:**
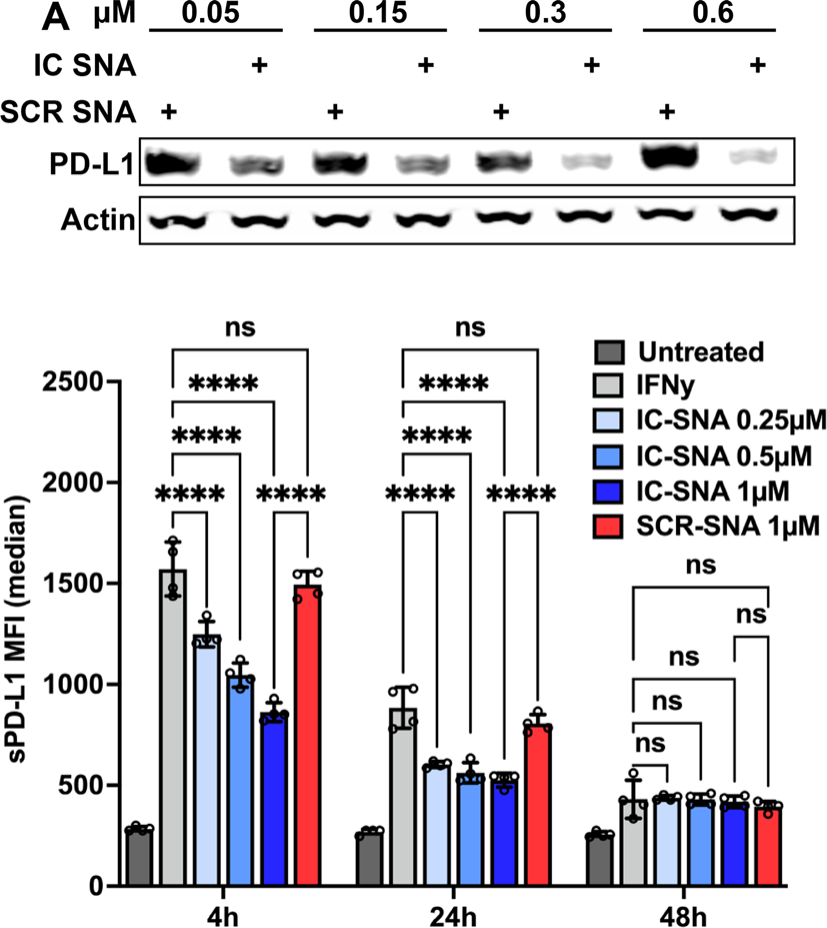
*In vitro* PD-L1 knockdown by IC-SNA treatment in MC38 colon cancer cells. (A) Dose-dependent knockdown of PD-L1 expression in MC38 cells by IC-SNAs. Actin was used as a standard control to ensure that an equal amount of protein lysates were loaded to each lane. (B) Surface PD-L1 (sPD-L1) expression levels following incubation with IC-SNAs as a function of DNA concentration and incubation time. The results are presented as the mean ± SD (*n* = 4). Statistical analysis was performed using one-way ANOVA. “**” *p* < 0.01; “***” *p* < 0.001; and “****” *p* < 0.0001.

To prepare IC-SNAs, sequence B was anchored onto ∼50 nm liposomal cores (Figure S2A), and the resulting structures were characterized via dynamic light scattering (DLS) and ζ potential. Upon DNA loading (∼50 strands per particle, Figure S2B), the average increase in diameter was 6.4 nm, and the decrease in ζ potential was 16 mV (Figure S2C). With these materials in hand, the effect of IC-SNAs on PD-L1 expression was explored in the same cell line as a single-entity agents (transfection agents are not needed in this case); SNAs comprised of a scrambled version of sequence B (SCR-SNAs) was used as a control. Increasing the concentration of IC-SNAs (from 0 nM to 0.6 μM in terms of DNA concentration) resulted in an increase in overall PD-L1 knockdown from 0 to ∼83%. Conversely, the SCR-SNA control did not influence PD-L1 expression (Figure 2A), indicating that the knockdown observed is sequence specific.

To confirm that IC-SNAs induce PD-L1 knockdown in cells other than MC38, PD-L1 knockdown was also evaluated in B16.F10 melanoma cells. Consistent with previous results,^12,16,18,19,68−72^ PD-L1 knockdown by IC-SNAs in B16.F10 cells was also dose dependent, albeit to a lesser extent compared to in the MC38 cells (Figure S3).

Because the knockdown of protein expression by antisense DNA is a dynamic process and PD-L1 mainly functions by binding to its receptor PD-1, it is critical to evaluate both the onset and the duration for reducing functional PD-L1 (i.e., surface PD-L1 in this context). Toward this end, MC38 cells were stimulated with IFN-γ and then incubated with a set of concentrations of IC-SNAs, and the sPD-L1 protein expression levels were evaluated at 4, 24, and 48 h postincubation (Figure 2B). At both 4 and 24 h, a statistically significant reduction in sPD-L1 expression was observed (Figure 2B, Figure S5, Table S2). Specifically, at 4 and 24 h, treatment with the 1 μM sample (in terms of DNA) resulted in an average of 45.1% and 40.6% reduction in sPD-L1 expression, respectively, as compared to the untreated IFN-γ positive control (Figure S6, Table S2). Conversely, at 48 h, no difference in sPD-L1 expression was observed between the IC-SNA-treated group and the control groups, presumably due to the decrease in IFN-γ stimulation across all groups over time (Figures S6, Table S2).

### Immunotherapeutic Potency and *In Vivo* Antitumor Efficacy of IC-SNAs in MC38 Colon Cancer Cells

To determine the ability of IC-SNAs to silence PD-L1 expression and act as antitumor immunotherapeutics *in vivo*, C57BL/6 mice bearing MC38 colon cancer tumors (*n* = 4 per group) were subcutaneously administered IC-SNAs peritumorally every 2 days (20 nmole per injection in DNA amount) starting on day 7 until days 21–25 post-tumor inoculation. The results attained with these animals were compared to those attained with animals that were untreated or those that were treated with SCR-SNAs (negative controls).

**Figure 3:**
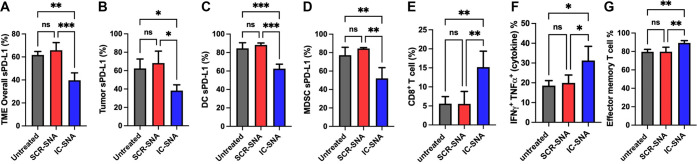
Influence of IC-SNA treatments on the TME. The expression levels of PD-L1 were measured by flow cytometry in different cell populations within MC38 tumors with treatment as indicated. A reduction in sPD-L1 expression was observed in the (A) total live cells of the TME, (B) tumor cells, (C) tumor-infiltrating DCs, and (D) tumor-infiltrating MDSCs. The results are presented as the mean ± SD (*n* = 3/4). (E) Percentage of tumor infiltrating CD8^+^ T cells. (F) Percentage of IFN-γ^+^ TNF-α^+^ cells among tumor-infiltrating CD8^+^ T cells. (G) Percentage of CD44^+^CD62L^–^ effector memory subset among tumor-infiltrating CD8^+^ T cells. Statistical analysis was performed using one-way ANOVA. “**” *p* < 0.01; “***” *p* < 0.001; and “****” *p* < 0.0001.

**Figure 4:**
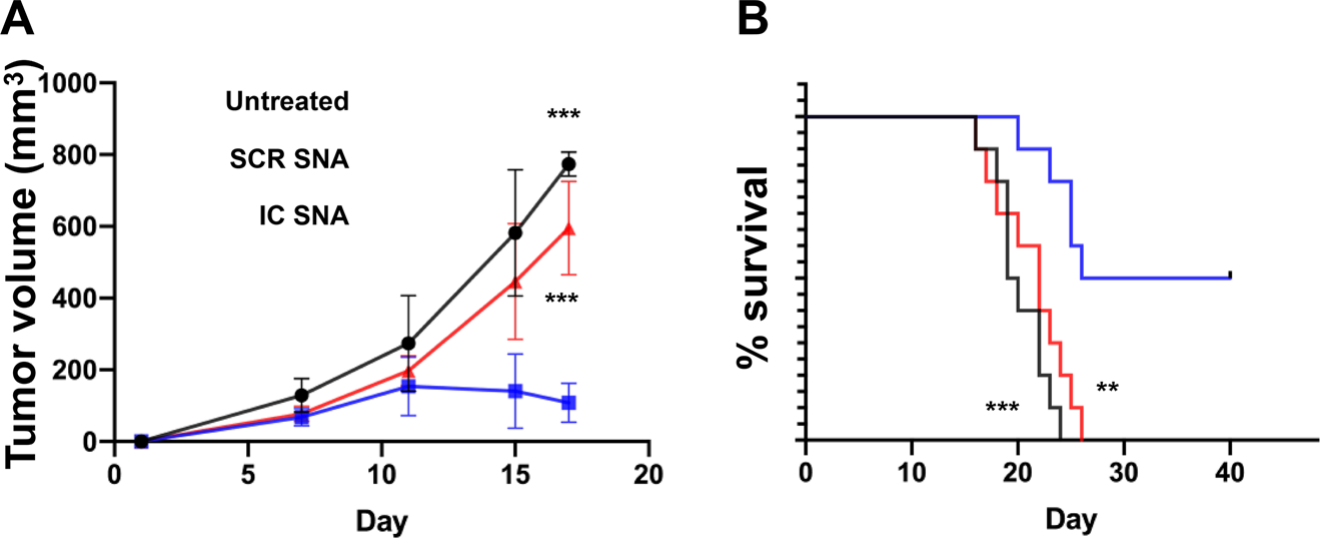
Antitumor efficacy of IC-SNAs in a MC38 mouse syngeneic tumor model. (A) Tumor growth (*n* = 4/group) and (B) survival analysis of MC38 tumor-bearing mice treated with IC-SNAs or SCR-SNAs, or untreated mice (*n* = 10/group). The results are presented as the mean ± SD. Statistical analysis was performed using one-way ANOVA (A) and a log-rank test for overall survival (B). “**” *p* < 0.01, and “***” *p* < 0.001.

At day 22, a subset of animals in each group were sacrificed and the tumor-infiltrating cells were stained and measured for sPD-L1 expression (Figure 3). IC-SNA treatment led to a decrease in overall PD-L1 expression within all live cells from tumor tissues, as compared to treatment with the SCR-SNAs and the untreated controls (Figure 3A, Figure S8A). Within individual cell populations, IC-SNAs reduced sPD-L1 expression in tumor cells (Figure 3B, Figure S8B), DCs (Figure 3C, Figure S8C), and MDSCs (Figure 3D, Figure S8D). Moreover, IC-SNAs enhanced antitumor T cell activity within the TME, as evidenced by increased: (1) accumulation of intratumoral CD8^+^ T cells (Figure 3E), (2) production of cytotoxic cytokines (IFN-γ and TNF-α) (Figure 3F), and (3) accumulation of an effector memory T cell (CD44^+^ and CD62L^–^) subset (Figure 3G). Importantly, IC-SNA treatment significantly reduced tumor growth (Figure 4A, Figures S10 and S11) and extended the survival of mice as compared to those animals that were treated with the scrambled SNAs and the untreated animals (Figure 4B), suggesting that the silencing PD-L1 by IC-SNAs contributes significantly to tumor clearance *in vivo*.

Finally, to preliminarily assess the efficacy of IC-SNAs relative to conventional antibody-based checkpoint inhibition, we completed a small pilot study (*n* = 4 per group) on the antitumor efficacy of IC-SNAs as compared to anti-PD-L1 antibodies. Significantly, IC-SNAs performed as well as antibody checkpoint inhibitors at suppressing tumor growth (Figure S12) relative to untreated controls. Because these agents act on the same target, but by completely different mechanisms of action, it is difficult to benchmark IC-SNAs against conventional antibody treatments. Nevertheless, this early pilot study provides direct evidence that IC-SNAs potentially can be used as an alternative to antibodies in cancer immunotherapy.

## Conclusion

This work shows that arranging antisense DNA on the nanoscale in the form of an SNA is a viable strategy for generating chemical constructs that act as potent immunotherapeutic agents. Furthermore, these data provide evidence that SNAs and gene knockdown pathways can be used as an alternative to traditional checkpoint inhibitors in cancer immunotherapy. This is an attractive approach to immunotherapy since sPD-L1 is not always a viable target because of the dynamic nature of protein expression and the ever-changing TME. Significantly, subcutaneous administration of IC-SNAs to mice bearing MC38 colon cancer tumors resulted in decreased PD-L1 expression in all cell types within the TME, increased T cell cytotoxicity, reduced tumor growth, and extended animal survival. Moreover, the data presented herein shows that SNAs can provide additional opportunities for regulating PD-L1, potentially with reduced side effects by virtue of peritumoral administration. In addition, since the SNA platform is scalable and modular, it establishes the potential for combining multiple antisense DNA and/or siRNA sequences directed toward different molecular targets (including multiple immune checkpoints) in the same cell on the same particle; thus, multiple immunosuppressive pathways can be silenced simultaneously. Taken together, this work highlights the promise of using nanostructured chemical constructs to regulate the action of PD-L1 on a genetic level and that this can be leveraged to yield powerful gene regulation agents for cancer immunotherapy.

## Supporting Information Available

Materials and methods, DNA design and binding accessibility profile, synthesis, and characterization of IC-SNAs, flow cytometry, *in vivo* experimental details and data (PDF)
