# Supplementary material for: Disrupting the Interplay between Programmed Cell Death Protein 1 and Programmed Death Ligand 1 with Spherical Nucleic Acids in Treating Cancer
Source: ACS Cent Sci. 2022 Aug 31;8(9):1299–305. doi: 10.1021/acscentsci.2c00717 (PMC9523766; doi:10.1021/acscentsci.2c00717)
Supplement: Supplementary file 1 — oc2c00717_si_001.pdf [file oc2c00717_si_001.pdf]

**SUPPORTING INFORMATION**

**for**

**Disrupting the Interplay Between Programmed Cell Death Protein 1 and Programmed Death Ligand 1 with Spherical Nucleic Acids in Treating Cancer**

Liyushang Chou[1,2], Cassandra E. Callmann[2,3], Donye Dominguez[4], Bin Zhang,[4, *] and Chad A. Mirkin[2,3,4,*]

[1]Interdisciplinary Biological Sciences Graduate Program, [2]International Institute for Nanotechnology, [3]Department of Chemistry, Northwestern University, Evanston, Illinois 60208, [4]Feinberg School of Medicine, Northwestern University, Chicago, Illinois, 60611, United States

Corresponding Author Emails: bin.zhang@northwestern.edu, chadnano@northwestern.edu

## *Table of Contents*

***Materials and Methods*** ............ ***4***

**Design of antisense sequences against mouse PD-L1 (mPD-L1)** ............ **4**

**DNA synthesis and purification** ............ **4**

**Liposome/IC-SNA synthesis** ............ **5**

**Western Blot Analysis** ............ **6**

**KD Flow cytometry analysis** ............ **7**

***In vivo* T cell function analysis** ............ **8**

**Safety statement** ............ **8**

............ *Error! Bookmark not defined.*

***Scheme S1. Antisense DNA design rationale.*** ............ ***9***

***Figure S1. Antisense DNA binding accessibility profile*** ............ ***10***

***Figure S2. Synthesis and characterization of IC-SNAs*** ............ ***11***

***Table S1. Antisense oligonucleotides sequences used*** ............ ***12***

***Figure S3. Western blot of PD-L1 knockdown by IC-SNAs in the B16F10*** ............ ***13***

............ *Error! Bookmark not defined.*

***Figure S4. Evaluation of PD-L1 Expression by Flow Cytometry.*** ............ ***14***

***Figure S5. Flow panels for time-dependent sPD-L1 reduction by IC-SNA treatments.*** ............ ***15***

***Table S2. MFI of sPD-L1 as a function of incubation time after IC-SNA treatment*** ............ ***16***

***Figure S6. Percentages of sPD-L1 knockdown (MFI) by IC-SNA treatments in RPMI-1640 compared to IFN-$\gamma$ positive controls at each time point.*** ............ ***17***

*Figure S7 Gating strategy to evaluate TME PD-L1 expression* ....................18

*Figure S8. Flow panels for TME PDL1 KD.* ....................19

A) TME overall sPDL1 ....................19

B) Tumor sPDL1 ....................20

D) Infiltrated MDSC sPDL1 ....................22

*Figure S9. Gating strategy to evaluate TME CD8+ T cells* ....................23

*Figure S10. Animal Dosing Schedule.* ....................24

*Figure S11. Individual tumor growth curves* ....................24

*Figure S12. Anti-tumor efficacy of IC SNA vs. Anti-PD-L1 antibody* ....................25

*References* ....................26

***Materials and Methods***

**Design of antisense sequences against mouse PD-L1 (mPD-L1)**

Antisense DNA sequences targeting mPD-L1 mRNA were not available in the literature. Therefore, sequences were designed for this study that are complementary to mouse PD-L1 mRNA. This sequence information was obtained from the publicly available NCBI GeneBank. To avoid non-specific, off-target effects, the mouse PD-L1 mRNA was checked for possible homology using a BLAST test. To achieve efficient gene knockdown, mRNA secondary structure was considered because it plays an important role in the accessibility of interacting exogenous oligonucleotides. To avoid binding regions with existing secondary structures, Mfold was used to simulate the possible ways in which the mRNA could fold. The antisense sequence was designed based on the following rules: a) the binding regions avoid translation machinery-occupied regions (the 3′ and 5′ ends); b) complementary sequences are free of secondary structures; c) antisense sequences are composed of 15 nucleotides with 35 % to 60 % GC content and melting temperatures ($T_m$s) of 50 °C. Four antisense oligonucleotide sequences were selected for evaluation in cells (Sequence A: 5'- AGT CCT TTG GAG CCG -3', Sequence B: 5'- TGA CGT TGC TGC CAT -3', Sequence C: 5'- AGC TGG TCC TTT GGC -3', Sequence D: 5'- GAT GTG TTG CAG GCA -3'). For the scrambled sequence, an online scrambler tool was used to scramble sequence B (SCR: 5′-GCT GTG CTA CTA TGC-3′).

**DNA synthesis and purification**

All sequences were synthesized with phosphorothioate backbones using standard automated solid-phase phosphoramidite DNA synthesis protocols on a MerMade 12 synthesizer (Bioautomation). Universal CPG solid supports and cholesterol-ended supports were used to synthesize linear sequences and cholesteryl-modified sequences, respectively. The automated solid-phase DNA synthesis uses 4,5-dicyanoimidazole as the activator and

3-(dimethylamino-methylidene amino)-3H-1,2,4-dithiazole-3-thione as the sulfurizing agent. The synthesized DNA strands were cleaved from the solid support via incubation in 30 % ammonium hydroxide at 55 °C overnight. Subsequently, excess ammonia was removed by evaporation under nitrogen gas at room temperature. Then, the oligonucleotides were dissolved in distilled water and filtered before purification. An Agilent high-pressure liquid chromatography (HPLC) system with a C4 or C18 column was used to purify cholesterol-ended and universal-ended sequences, respectively. Gradient triethylammonium acetate and acetonitrile (10 % to 100 % acetonitrile) was used to remove failure strands over 30 min. The purified oligonucleotides were concentrated and lyophilized. The powdered oligonucleotides were incubated with 20 % acetic acid at room temperature for 1 h and then extracted three times using an equal volume of ethyl acetate. The deprotected DNA was lyophilized and reconstituted in 1 mL of deionized water. The sequences were characterized using matrix-assisted laser desorption ionization time-of-flight (MALDI-ToF).

**Liposome/IC-SNA synthesis**

Five mg of 1,2-dioleoyl-sn-glycero-3-phosphocholine (DOPC) powder was resuspended in 5 mL of phosphate-buffered saline (PBS) solution. Liposomes were formed via 5 freeze-thaw cycles, where liquid nitrogen and sonication in a 37 °C water bath were used. The liposomes were then extruded sequentially through polycarbonate filters (T&T Scientific Corp., Knoxville, TN): 200 nm, 100 nm, 80 nm, and finally 50 nm in filter pore size. The liposome concentration was measured using a commercially available phosphatidylcholine assay (Sigma). Cholesterol-ended oligonucleotides were inserted into the double layer of the phospholipid membranes via hydrophobic interactions. Sodium dodecyl sulphate–polyacrylamide gel electrophoresis (SDS PAGE) was run to estimate the average number of strands that could be anchored on a 50-nm liposome (an average of 50 strands per particle

was observed). To form SNAs, oligonucleotides were added to liposomes (75:1), and the sample was shaken overnight at 35 °C. The oligonucleotide concentration was determined by UV-vis absorption at 260 nm (Figure S1). The resulting SNAs were then concentrated to 50 μM by DNA concentration using centrifugation filter units (Millipore), which also removed any unbound DNA. The resulting structures were analyzed to determine their zeta potential and size (Malvern Zetasizer) and DNA loading (gel electrophoresis).

**Western Blot Analysis**

MC38 cells were seeded in six-well plates at 5 $x10^5$ cells per well overnight, and subsequently were stimulated with IFN-γ (20 ng/mL in culture media) for 24 h. The cell lysates were harvested 48 h after transfection with the free oligonucleotide (and Lipofectamine 2000). For SNA treatments (IC-SNAs or scrambled SNAs), MC38 cells and B16f10 cells were seeded in six-well plates at 5 $x10^5$ cells per well overnight, subsequently were treated with either IC-SNAs or scrambled SNAs at different DNA concentrations for 24h. Subsequently cells were washed with PBS stimulated with IFN-γ (20 ng/mL in culture media) for 24 h before harvesting. The cell lysates were measured and diluted so that an equal amount of protein lysates was loaded into each lane on the 4%–12% SDS-PAGE (Life Technologies). The separated protein was then transferred via the iBlot™ 2 Gel Transfer system (Invitrogen). The blotted membrane was then washed with PBS and blocked with 5 % milk in phosphate buffered saline with 0.1% Tween 20 (PBS/Tween) for 1 h, and then stained with the iBind Automated Western System (Thermofisher). The antibodies used were: 1 μg/mL PD-L1 antibody (abcam), 0.5 μg/mL beta actin (abcam), and 2 μg/mL IRDye 800CW donkey anti-rabbit secondary antibody (LI-COR). After 3 h of staining, the incubated membrane was washed three times in 1× PBS and imaged on an Odyssey CLx system (LI-COR).

**Knockdown Flow cytometry analysis**

MC38 cells were seeded on 24-well plates at 5 x$10^4$ cells per well overnight, and they were subsequently stimulated with IFN-$\gamma$ (20 ng/mL culture media concentration) for 24 h on the following day to spur PD-L1 expression. The cells were treated with either IC-SNAs or scrambled SNAs at a concentration gradient in Opti-MEM media for 6 h before washing and resubstituting with fresh RPMI-1640 media for longer incubation (24 h and 48 h). The cells were harvested and stained for surface PD-L1 4 h, 24 h, and 48 h after treatment. In every batch, duplicates of untreated and IFN-$\gamma$ stimulated samples stained without anti-PD-L1 served as the unstained FMO control.

For the antitumor efficacy studies, C57Bl/6J mice were utilized (Jackson Labs). On day 0, ~1x$10^6$ MC38 tumor cells were injected subcutaneously into the right flank. Treatments commenced on day 7, once tumors were established (30-50 $mm^3$ in size). Each group (n=4) were administered either IC-SNA or SCR SNA (both 20 nmol with respect to DNA) peritumorally every 48 hours. On day 22, the mice were sacrificed and tumors harvested to evaluate the *in vivo* PD-L1 knockdown. The collected tumor tissue was digested in a solution of RPMI-1640 with collagenase IV (5 mg/ml) and deoxyribonuclease (DNase, 50 units/ml) at 37° C for 1 h and filtered through a 40-μm cell strainer. Cells were centrifuged and resuspended in red blood cell lysis buffer for 10 minutes, followed by another round of centrifugation to neutralize and remove lysis buffer. [1]

Every batch of cells was stained for viability and surface PD-L1. In particular, the LIVE/DEAD™ Fixable Dead Cell Stain Kit was used according to the manufacturer's protocol. In short, fluorescent reactive dye was reconstituted with 50 μL of dimethyl sulfoxide (DMSO) and diluted to 1:1,000 dye in the staining buffer. The cells were incubated with staining buffer for 30 min at 4 °C. The cells were then centrifuged down, and the supernatant was removed. From here, the cells were stained for surface PD-L1. To stain surface PD-L1, diluted anti-PD-

L1 (BioLegend) (0.5 μL per sample) was added to flow tubes for a 20-min incubation on ice in the dark. Afterwards, the cells were washed with cell staining buffer and centrifuged down for another 20 min of incubation at room temperature with fixation buffer (BioLegend). MFI was used to measure PD-L1 expression. *For in vivo* staining, cells were also stained as described above and gated on CD45 to analyze tumor (CD45-) and immune infiltrates (DC: CD45+, CD11b/c +, MDSC: CD45+, CD11b+,GR1+) KD respectively. PD-L1 expression level is measured by gating according to the FMO control of a specific population

### *In vivo* T cell function analysis

Cohorts of five C57Bl/6J female mice per group were inoculated with ~1x$10^6$ MC38 cells subcutaneously in the right flank. Treatments started on day 7-9 when tumors reached an average volume of 30- 50 $mm^3$. IC-SNAs and SCR SNAs were injected peritumorally (n=4/group, 20 nmol with respect to DNA) every two days until day 21-25. Tumor samples were harvested and processed into a single cell suspension as described previously. For cytokine analysis, the tumor samples were restimulated using a standard protocol [2]. For analysis of activation markers, CD45+ CD8+ TCRβ+ cells were selected. Intracellular IFN-γ and TNF-α was measured to evaluate T cell cytokine release. CD44 and CD62L were stained to detect effector memory T cell subset accordingly.

### Safety statement

No unexpected or unusually high safety hazards were encountered.

***Scheme S1. Antisense DNA design rationale.***

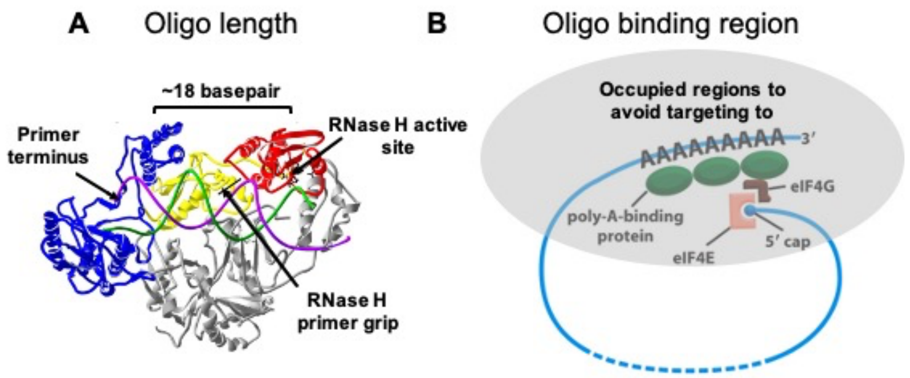


A) Oligonucleotides can be designed to trigger RNase H-mediated DNA:RNA hybrid degradation. In this case, the sequences were designed to fit into the active cleavage site of the RNase H enzyme. All sequences were 18 bp or less in length. B) The other mechanism of disrupting protein expression with antisense DNA occurs on a translational level, where antisense DNA binds to mRNA and halts the translation machinery. To employ this mechanism, the sequences were designed to avoid binding to preoccupied regions during translation (the gray area).

***Figure S1. Antisense DNA binding accessibility profile.***

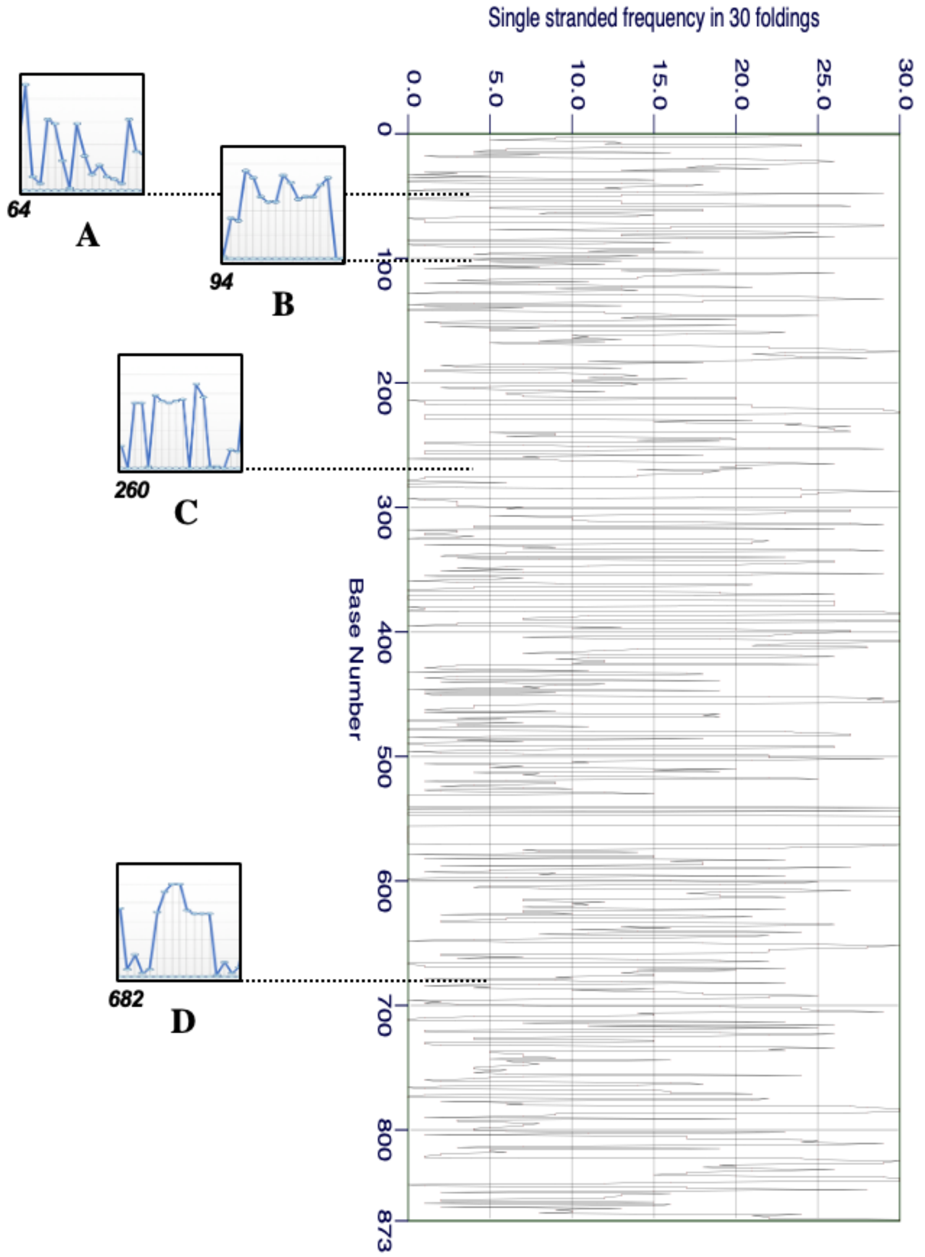


The coding sequence (CDS, starts at 84 bp and ends at 956 bp) of mPD-L1 mRNA was simulated to fold using the online Mfold RNA folding tool. The overall length of this segment is 873 bp. The horizontal parameter depicts the single-stranded frequency for a given bp position in 30 simulated folds. Four candidate sequences were synthesized based on the availability of a given 15-bp segment that has a strong binding ability in the middle section.

*Figure S2. Synthesis and characterization of IC-SNAs*

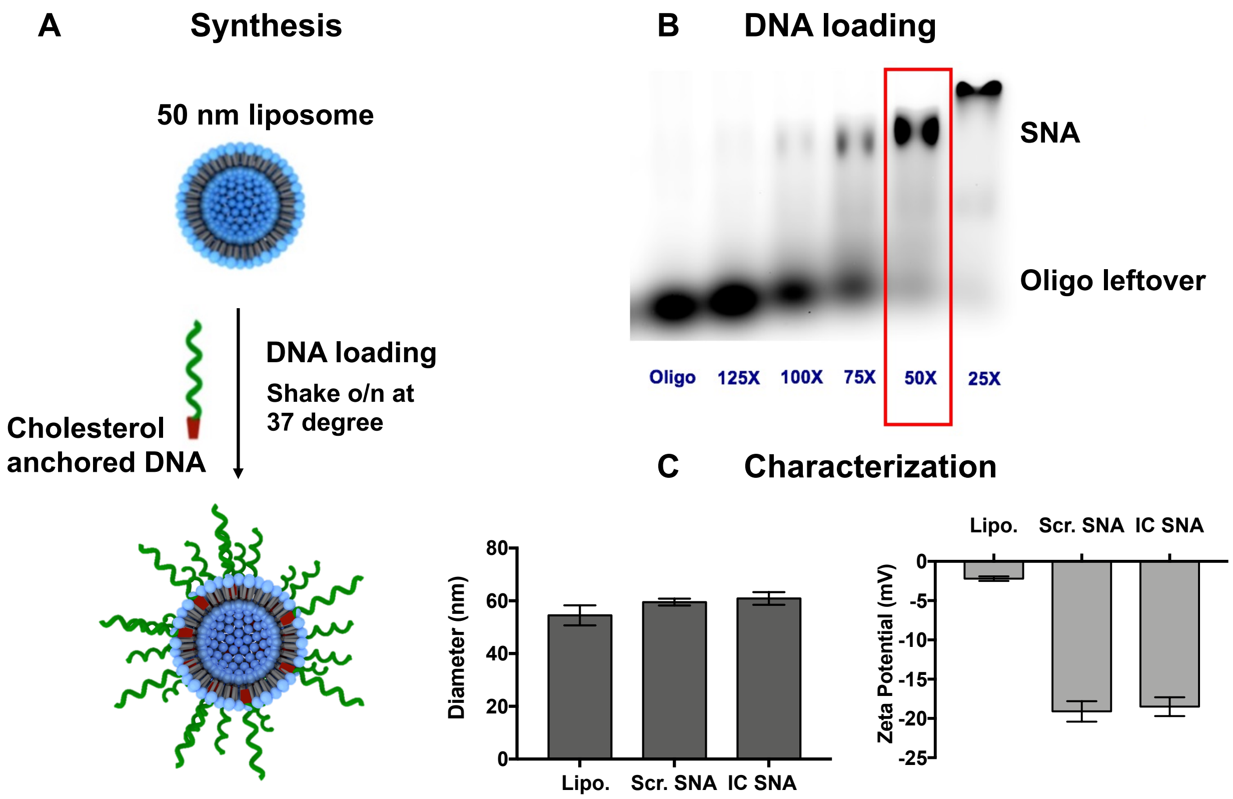


A) SNAs were synthesized using Sequence B and characterized using Matrix-Assisted Laser Desorption Ionization—Mass Spectrometry (MALDI-MS). B) Subsequently, the SNA was characterized using polyacrylamide gel electrophoresis, C) dynamic light scattering (DLS), and zeta potential. The loading for dye-labeled DNA-based SNAs was measured using electrophoresis. On average, the SNAs were modified with 50 strands per particle. Upon DNA loading, the average increase in diameter was 6.4 nm, and the decrease in zeta potential was 16 mV.

| Sequence purposes | Synthesized sequences |
|---|---|
| **Targeting PD-L1 sequences for transfection** | **(A) 5'- AGT CCT TTG GAG CCG -(spacer18)2-TT-3',<br>(B) 5'- TGA CGT TGC TGC CAT -(spacer18)2-TT-3'<br>(C) 5'- AGC TGG TCC TTT GGC -(spacer18)2-TT 3'<br>(D) 5'- GAT GTG TTG CAG GCA --(spacer18)2-TT 3'** |
| **Targeting PD-L1 sequence for ICSNA anchorage** | **(B) 5'- TGA CGT TGC TGC CAT -(spacer18)2-cholesterol-3'** |
| **Non targeting control sequence for scramble SNA anchorage** | **Scramble (B) 5'-GCT GTG CTA CTA TGC-(spacer18)2-cholesterol-3'** |

***Table S1. Antisense oligonucleotides sequences used***: three types of PS backboned antisense DNA sequences were used in this study (linear sequences for transfection, cholesterol-ended sequences for SNA synthesis)

***Figure S3. Western blot of PD-L1 knockdown by IC-SNAs in the B16F10.*** A concentration-dependence was observed, albeit to a lesser extent in this cell line.

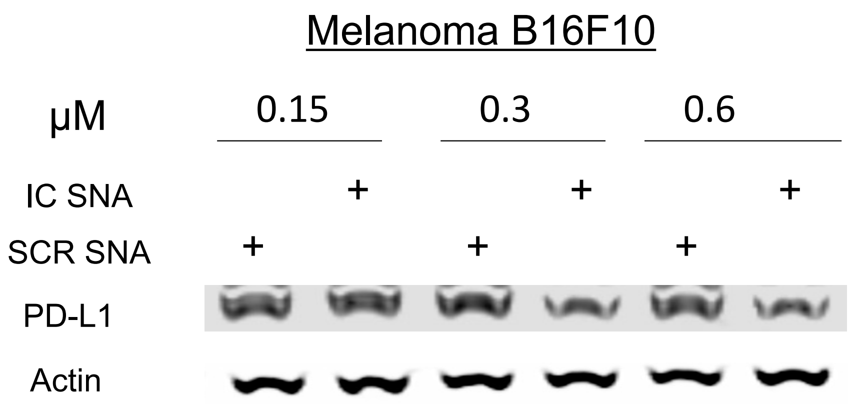

*Figure S4. Evaluation of PD-L1 Expression by Flow Cytometry.*

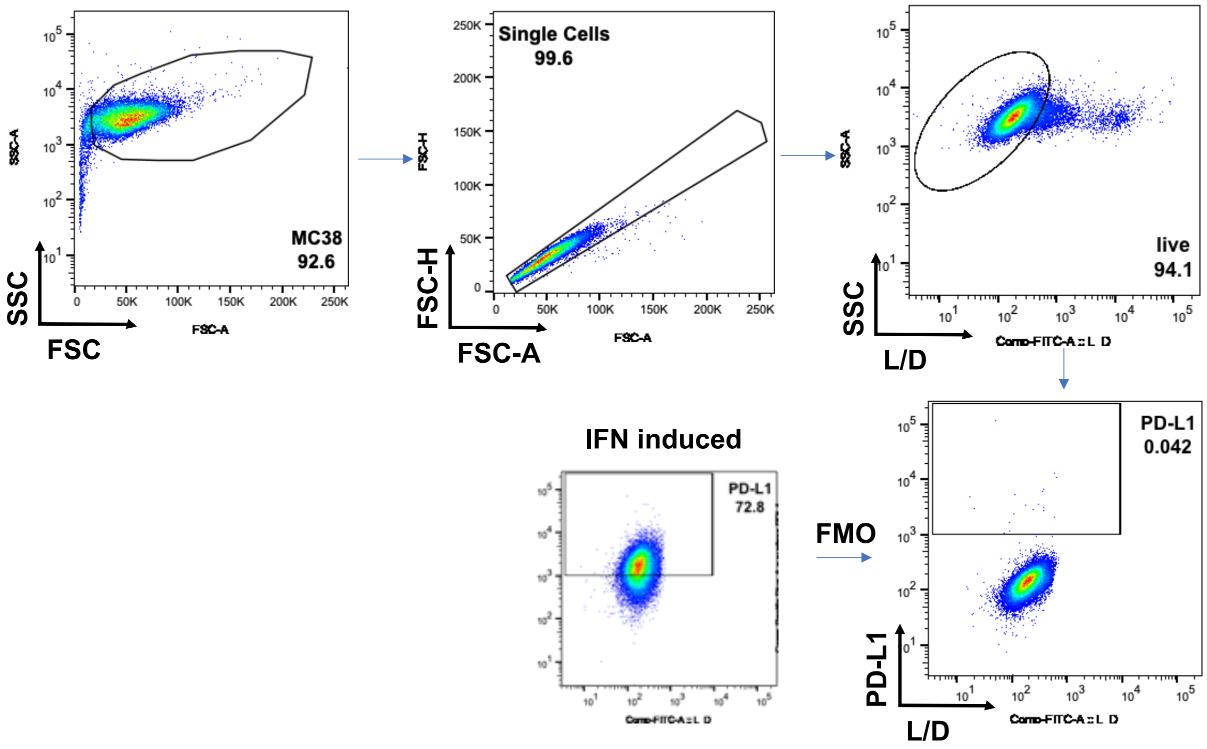


To analyze PD-L1 expression, cells were harvested and stained as described in the flow cytometry analysis section. One of the samples from untreated animals were stained without anti-PD-L1 antibody and used as the unstained control to establish the baseline in gating. The cells were gated by forward scatter (FSC) versus side-scatter (SSC) first, then gated on singlet cells, viability, then PD-L1 Fluorescence Minus One (FMO) control sample to establish the PD-L1 baseline.

***Figure S5. Flow panels for time-dependent sPD-L1 reduction by IC-SNA treatments.***

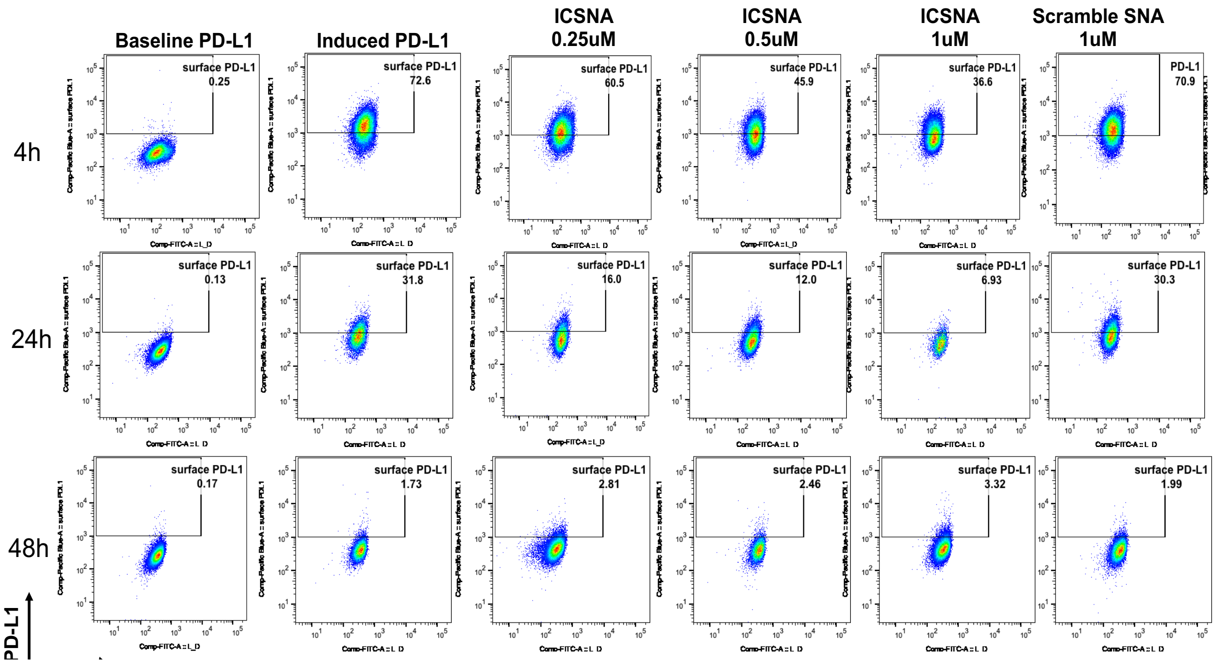

*Table S2. MFI of sPD-L1 as a function of incubation time after IC-SNA treatment.*

| | Untreated | IFNy | ICSNA 0.25μM | ICSNA 0.5μM | ICSNA 1μM | Scramble SNA 1μM |
|---|---|---|---|---|---|---|
| 4h sPD-L1 MFI | 274 | 1478 | 1227 | 1056 | 820 | 1422 |
| | 275 | 1438 | 1224 | 1123 | 930 | 1554 |
| | 299 | 1657 | 1204 | 980 | 853 | 1450 |
| | 285 | 1713 | 1341 | 1027 | 851 | 1546 |
| 24h sPD-L1 MFI | 275 | 816 | 586 | 558 | 475 | 807 |
| | 275 | 780 | 620 | 634 | 545 | 867 |
| | 247 | 964 | 599 | 532 | 539 | 763 |
| | 275 | 980 | 609 | 525 | 544 | 785 |
| 48h sPD-L1 MFI | 255 | 561 | 428 | 444 | 448 | 357 |
| | 275 | 424 | 443 | 460 | 436 | 395 |
| | 249 | 334 | 428 | 407 | 390 | 398 |
| | 244 | 404 | 452 | 406 | 398 | 419 |

***Figure S6. Percentages of sPD-L1 knockdown (MFI) by IC-SNA treatments in RPMI-1640 compared to IFN-γ positive controls at each time point.***

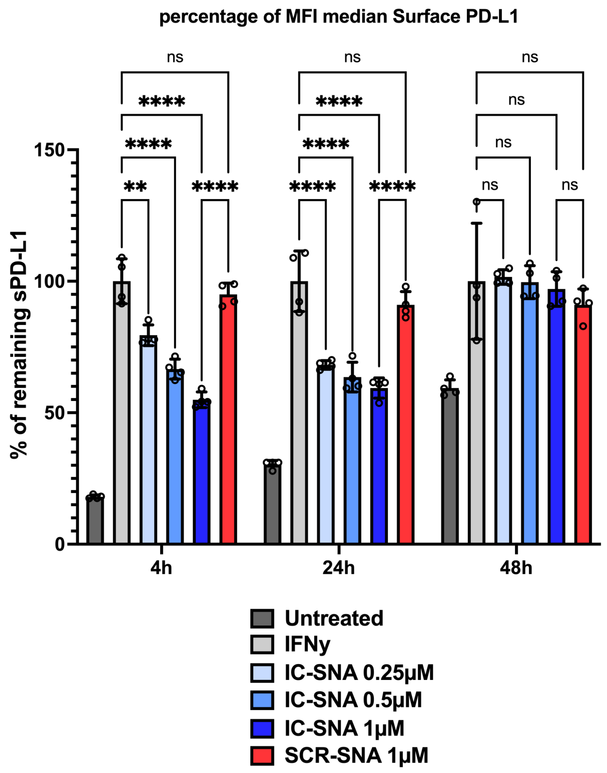

*Figure S7 Gating strategy to evaluate TME PD-L1 expression*

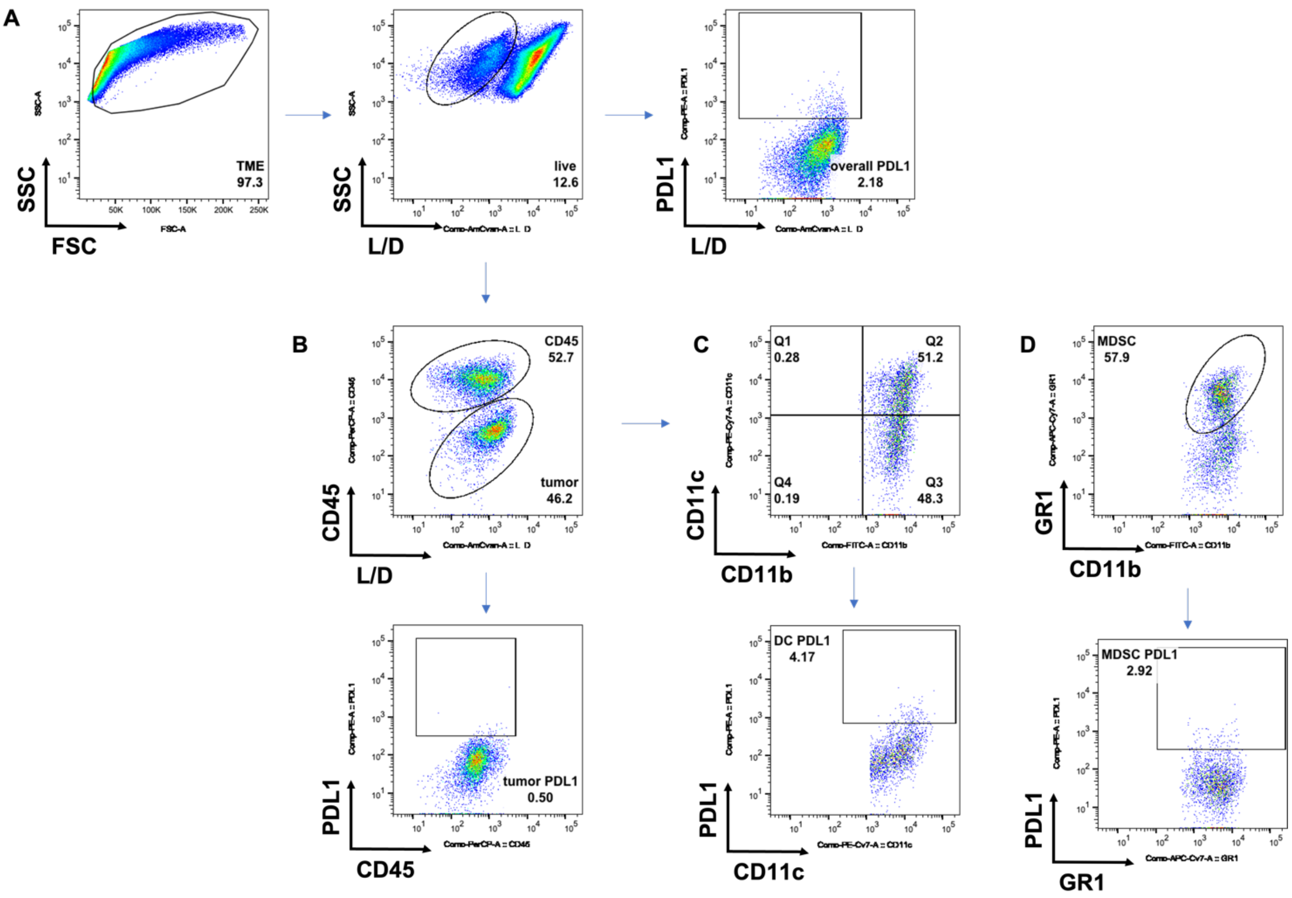


To analyze PD-L1 expression in the TME, cells were harvested and stained as described in the flow cytometry analysis section. One of the samples from untreated animals were stained without anti-PD-L1 antibody and used as the unstained control to establish the baseline in gating. A) The cells were gated by forward scatter (FSC) versus side-scatter (SSC) first, then gated on viability, then PD-L1 for overall PD-L1 in the TME. B) Tumor PD-L1 was measured by gating on CD45- cells. C) CD11b+, CD11c+ cells were gated to measure DCs PD-L1 expression. D) CD11b+, GR1+ cells were gated to measure MDSCs PD-L1 expression.

*Figure S8. Flow panels for TME PDL1 KD.*

A) TME overall sPDL1

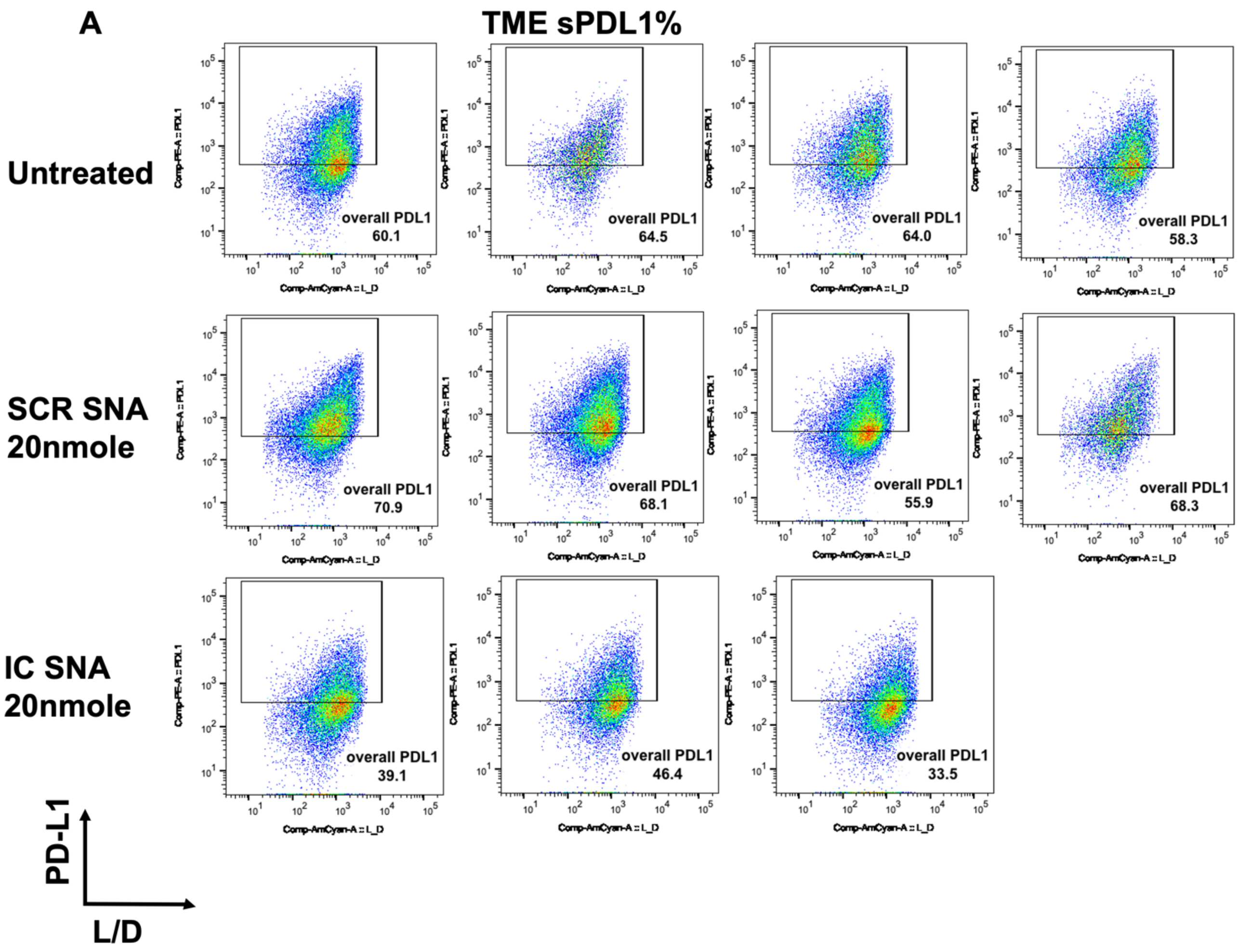

B) Tumor sPDL1

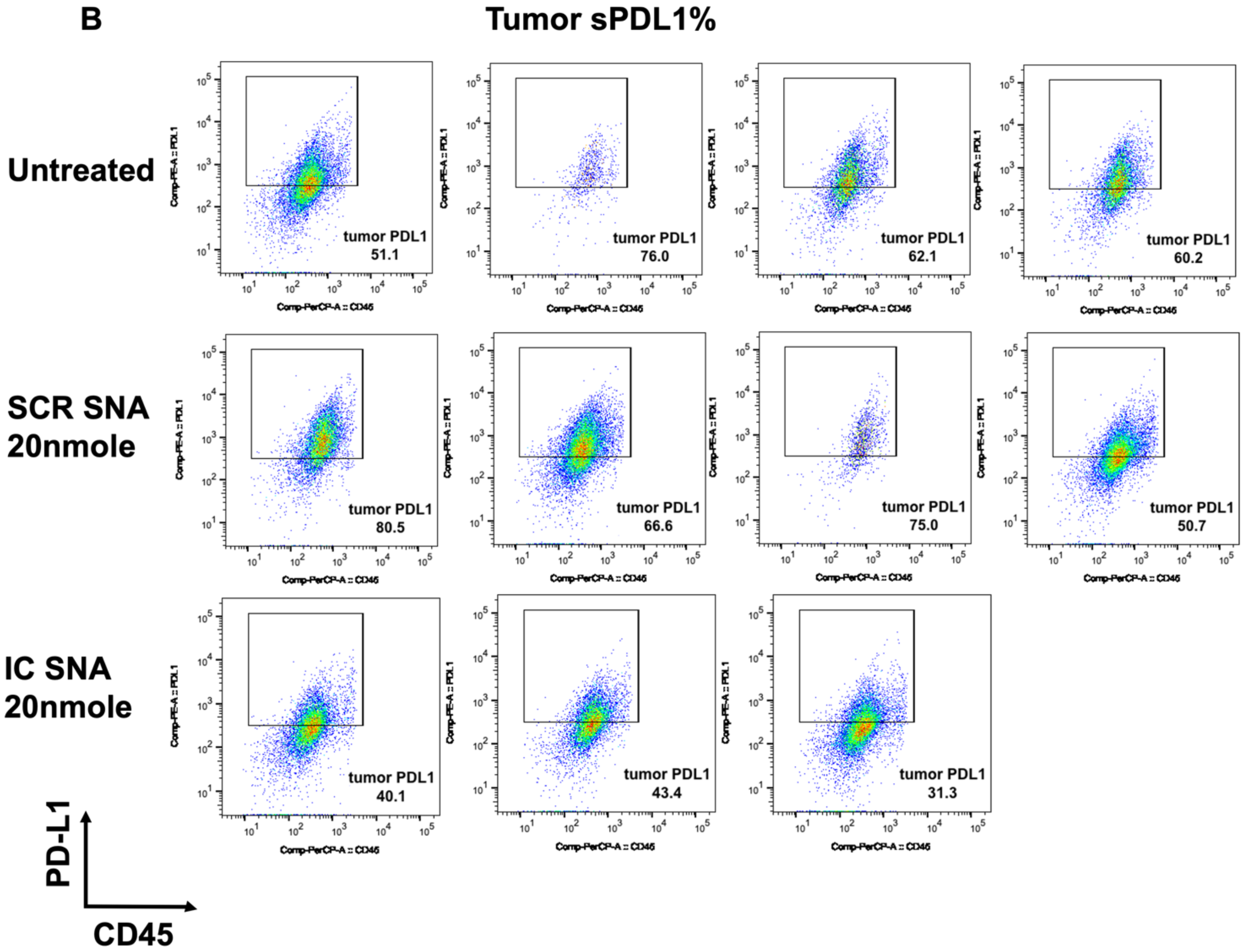

C) DC sPDL1

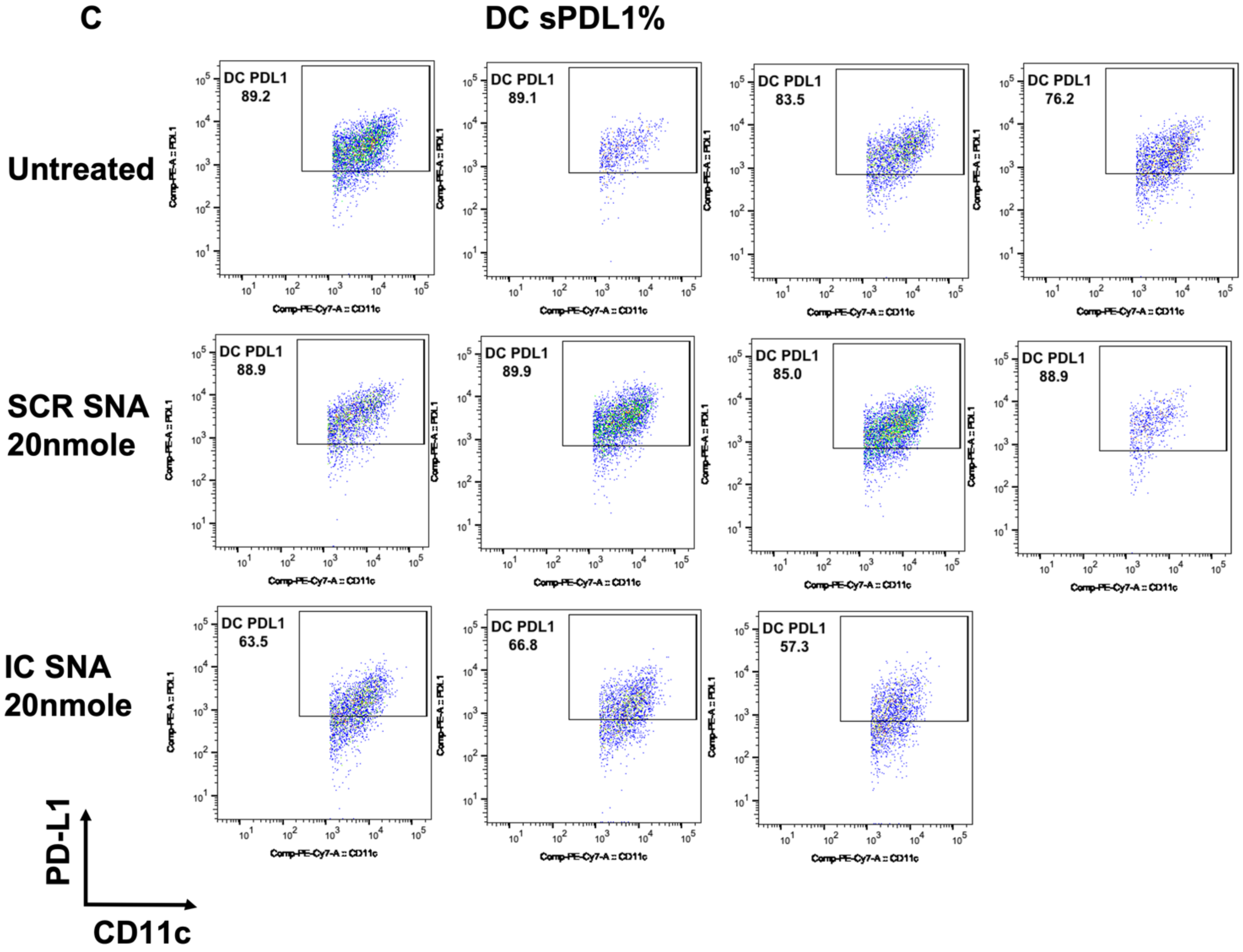
C
DC sPDL1%
Untreated
SCR SNA 20nmole
IC SNA 20nmole
DC PDL1
89.2
DC PDL1
89.1
DC PDL1
83.5
DC PDL1
76.2
DC PDL1
88.9
DC PDL1
89.9
DC PDL1
85.0
DC PDL1
88.9
DC PDL1
63.5
DC PDL1
66.8
DC PDL1
57.3
Comp-PE-A :: PDL1
Comp-PE-Cy7-A :: CD11c
PD-L1
CD11c

D) Infiltrated MDSC sPDL1

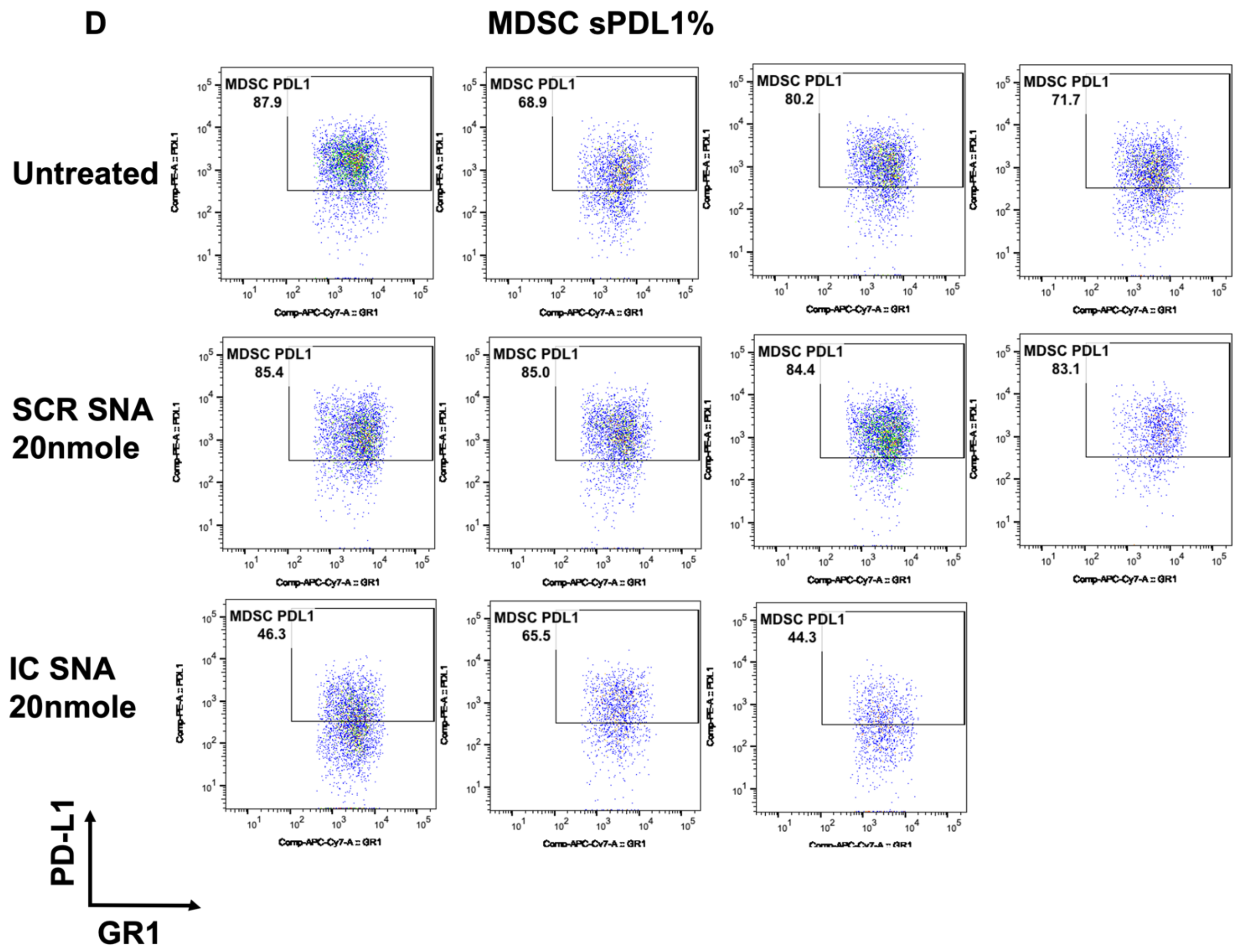

*Figure S9. Gating strategy to evaluate TME CD8+ T cells.*

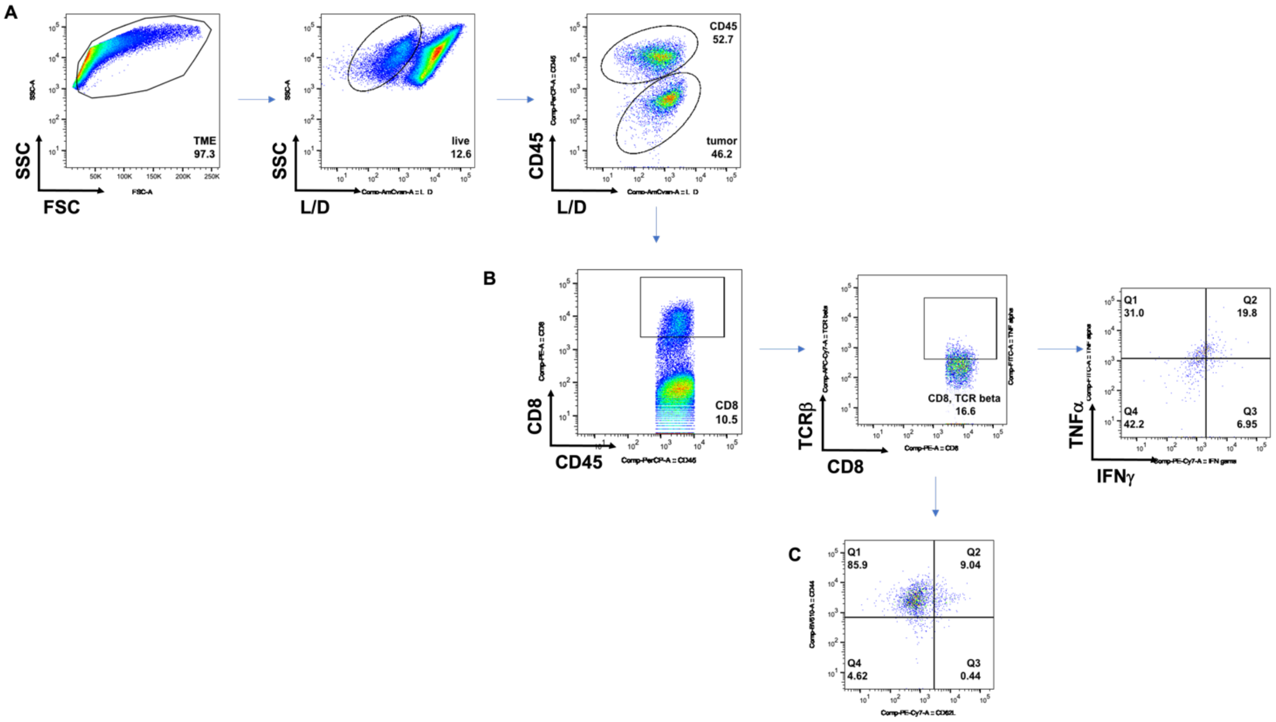


To analyze CD8 cells in the TME, cells were harvested and stained as described above in the flow cytometry analysis section. A) The cells were gated by forward scatter (FSC) versus side-scatter (SSC) first, then gated on viability, then CD45+ CD8+ TCRβ+ cells. B) To assess cytokine release, double positive population of IFNγ and TNFα were gated. C) Effector cells population were gated on CD44+ CD62L-.

***Figure S10. Animal dosing schedule.***

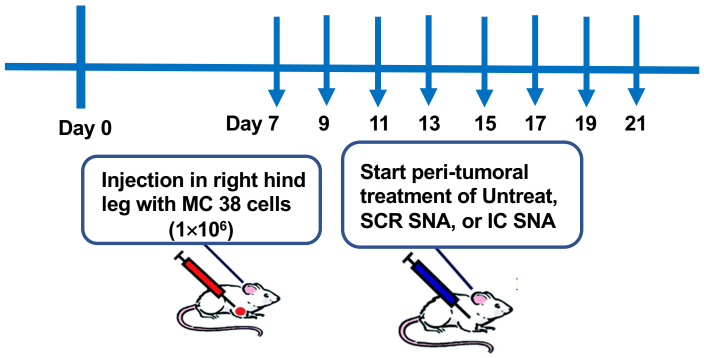


Mice were implanted with ~1x10$^6$ MC38 cells on Day 0. On day 7, mice in each group were peritumorally administrated IC-SNAs or SCR-SNAs every other day for minimum 8 treatments before sacrificing (n=4/group, 20 nmol with respect to DNA).

***Figure S11. Individual tumor growth curves*.**

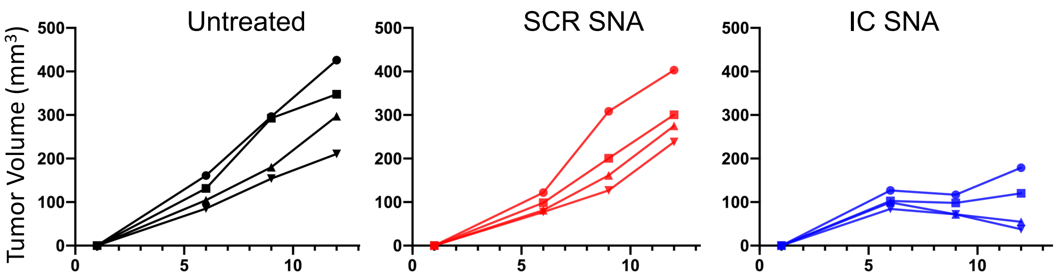

***Figure S12. Anti-tumor efficacy of IC SNA vs. Anti-PD-L1 antibody***

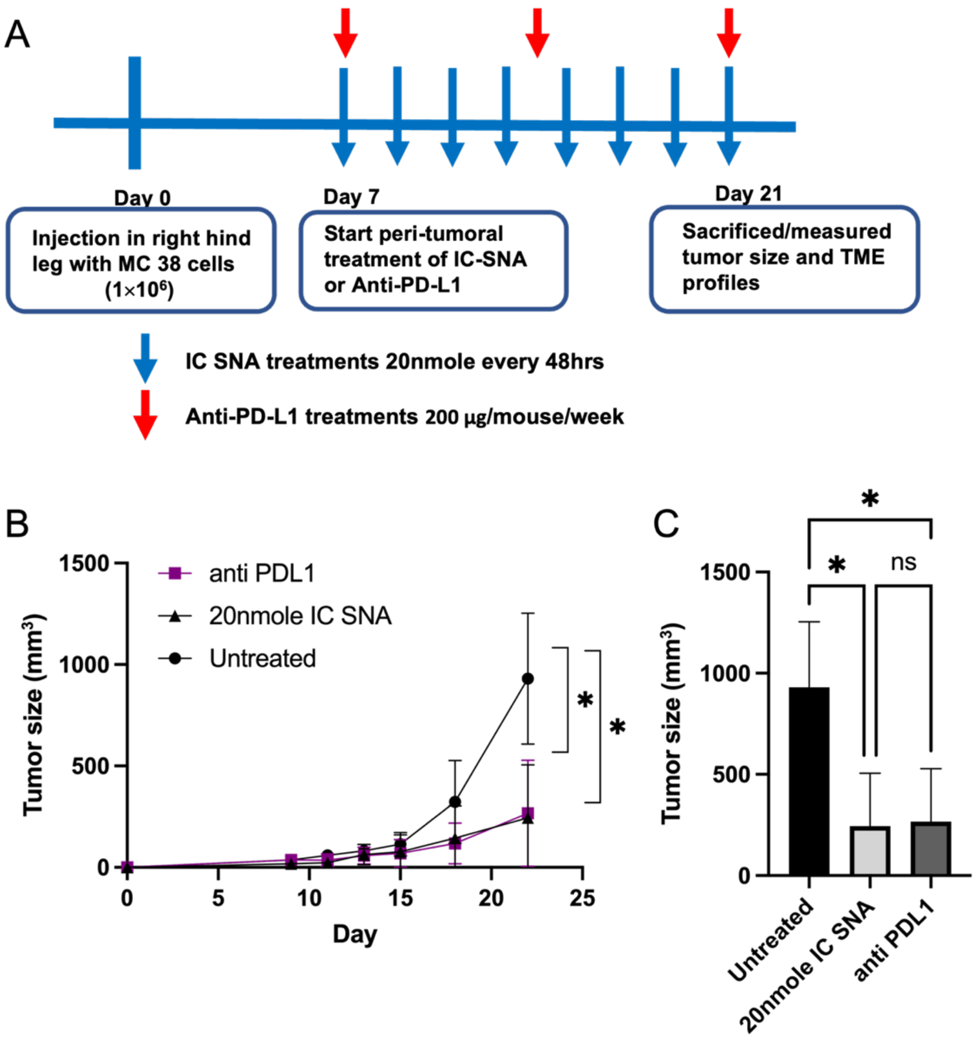


A) Mice were inoculated with ~1x10$^6$ MC38 cells in the right flank on Day 0. On day 7, IC-SNAs were administered peritumorally (n=4, 20 nmol with respected to DNA) every 48 for a total of 8 treatments. To compare IC-SNAs to antibody checkpoint inhibitors, a second cohort of animals (n=4) were administered 200 $\mu$g of anti-PD-L1 antibody were administrated peritumorally every 7 days for a total of 3 treatments based on precedence in the Zhang Lab. B) Tumor growth curve of animals administered anti-PD-L1 (purple square), IC-SNAs (grey triangle), and untreated controls (black circle). C) Tumor volume of each treatment group on Day 22.

### *References*


1 Zhao, X., Li, L., Starr, T. K. & Subramanian, S. Tumor location impacts immune response in mouse models of colon cancer. *Oncotarget* **8**, 54775-54787, doi:10.18632/oncotarget.18423 (2017).

2 Mandala, W., Harawa, V., Munyenyembe, A., Soko, M. & Longwe, H. Optimization of stimulation and staining conditions for intracellular cytokine staining (ICS) for determination of cytokine-producing T cells and monocytes. *Current Research in Immunology* **2**, 184-193, doi:https://doi.org/10.1016/j.crimmu.2021.10.002 (2021).
